# Diversity of laccase-coding genes in *Fusarium oxysporum* genomes

**DOI:** 10.3389/fmicb.2015.00933

**Published:** 2015-09-10

**Authors:** Natalia Kwiatos, Małgorzata Ryngajłło, Stanisław Bielecki

**Affiliations:** Institute of Technical Biochemistry, Faculty of Biotechnology and Food Sciences, Lodz University of TechnologyLodz, Poland

**Keywords:** laccase, multicopper oxidase, *Fusarium oxysporum*, pathogen, *in silico* modeling

## Abstract

Multiple studies confirm laccase role in fungal pathogenicity and lignocellulose degradation. In spite of broad genomic research, laccases from plant wilt pathogen *Fusarium oxysporum* are still not characterized. The study aimed to identify *F. oxysporum* genes that may encode laccases *sensu stricto* and to characterize the proteins *in silico* in order to facilitate further research on their impact on the mentioned processes. Twelve sequenced *F. oxysporum* genomes available on Broad Institute of Harvard and MIT (2015) website were analyzed and three genes that may encode laccases *sensu stricto* were found. Their amino acid sequences possess all features essential for their catalytic activity, moreover, the homology models proved the characteristic 3D laccase structures. The study shades light on *F. oxysporum* as a new source of multicopper oxidases, enzymes with possible high redox potential and broad perspective in biotechnological applications.

## Introduction

*Fusarium oxysporum* is a known plant wilt pathogen that attacks various crops such as corn, tomato, or banana. Most of the research involving these ascomycetes is focused on understanding of its virulence and prevention of plants’ devastation. The Broad Institute proceeds with *Fusarium* Comparative project and now 12 genomes of sequenced *F. oxysporum* strains are available on their website. Despite of broad genomic studies, still not much is understood about its proteome and in particular about its secreted enzymes. Extracellular laccases are one of the factors pointed out to be responsible for fungal virulence, however, so far only single genes were investigated, but now, with so many genomes available, broader analysis is possible (Ma et al., 2010; Broad Institute of Harvard and MIT, 2015).

Laccases (EC 1.10.3.2) are oxidoreductases from multicopper oxidases family that catalyze 4-electron reduction of O_2_ to water with simultaneous oxidation of organic substrates. These multicopper oxidases possess wide range of specificity and are able to oxidize both phenolic substances and non-phenolic ones with presence of mediators. Nowadays laccases are one of the most desired biocatalysts – they catalyze wide range of chemical reactions, thus can be applied in various industrial areas – pulp and paper, textile, food industries, diagnostics, waste water treatment, and chemical compounds synthesis. Their environmental friendly nature continuously focuses attention on the enzyme (Alcalde, 2007; Kunamneni et al., 2008; Madhavi and Lele, 2009; Giardina et al., 2010; Pezzella et al., 2015).

There are certain characteristics that distinguish laccases from other multicopper oxidases. Firstly, they must contain multicopper oxidase domains (Pfam: PF00394, PF07731, and PF07732), in fungi most of them contain all three domains. Moreover, signatures of laccases were distinguished – four 8–24 amino acid sequences characteristic for these proteins, called L1, L2, L3, and L4. These signatures comprise amino acids that coordinate copper atoms in T1 and T2/T3 centers. Two serines and one arginine in the signatures form SDS gate, a channel responsible for proton transfer. What is more, it is said that the laccases from ascomycetes should contain DSG[ILV] on the C-terminus (Kumar et al., 2003; Cázares-García et al., 2013).

In the past, 15 potential laccase genes were discovered in *F. oxysporum* f. sp. *lycopersici* 4287 and few of them studied in the aspect of pathogenicity (Cañero and Roncero, 2008; Reyes-Medina and Macías-Sánchez, 2015). Our study aimed to identify and analyze potential laccases *sensu stricto* in all sequenced *F. oxysporum* strains. Redundancy analysis of all multicopper oxidases was done and sequence, structure, and phylogenetic studies were performed on the chosen proteins.

## Materials and Methods

### Redundancy Analysis

All DNA and protein sequences used in the study were obtained from “*Fusarium* Comparative Sequencing Project, Broad Institute of Harvard and MIT (2015). The proteins that possess Cu-oxidase, Cu-oxidase2, or Cu-oxidase3 (Pfam: PF00394, PF07731, and PF07732) were selected using tools available on the website. The protein sequences were downloaded and subjected to further analysis (Step 1). ScanProsite was used to select proteins with certain sequence features (de Castro et al., 2006). In the following step (Step 2), Prosite patterns and L1 and L3 signatures were found. In the third step, only those proteins who possess all four L1–L4 signatures and MCO1 and MCO2 patterns were chosen (Step 3). **Table 1** gathers the patterns used for our search.

**Table 1:** Characteristic sequence motifs.

Characteristic sequence	
Cu-oxidase, Cu-oxidase2, Cu-oxidase3 domain	Pfam: PF00394, PF07731, and PF07732
Prosite patterns: PS00079, PS00080	G-x-[FYW]-x-[LIVMFYW]-x-[CST]-x-{PR}-{K}-x(2)-{S}-x-{LFH}-G-[LM]-x(3)-[LIVMFYW], H-C-H-x(3)-H-x(3)-[AG]-[LM]
L1	H-W-H-G-x(9)-D-G-x(5)-Q-C-P-I
L2	G-T-x-W-Y-H-S-H-x(3)-Q-Y-C-x-D-G-L-x-G-x-[FLIM]
L3	H-P-x-H-L-H-G-H
L4	G-[PA]-W-x-[LFV]-H-C-H-I-D-A-E-x-H-x(3)-G-[LMF]-x(3)-[LFM]
C-terminus	D-S-G[LIV]

All protein sequences in the sequential steps were divided into groups on the basis of sequence identity. The member of a given group must have been identical in more than 95%. It was assessed by multiple sequence alignment, tree building and identity analysis. The following tools were used: ClustalW2, Mega6, sequence identity and similarity (SIAS), interactive Tree Of Life (iTOL), and CLC Sequence Viewer.

### Analysis of Gene and Protein Sequences

Gene localization on chromosomes were visualized by Ensembl website tools.

Subcellular localization of proteins were predicted by SignalP 4.0, TMHMM2.0, and Phobius (default settings; Käll et al., 2004; Petersen et al., 2011; TMHMM Server, v. 2.0, 2015). Isoelectric points and molecular weight were analyzed by ExPASy – Compute pI/Mw Tool (2015). NetNGlyc 1.0 Server (2015; ExPASy – Compute pI/Mw Tool, 2015) was used to find possible glycosylation sites. The localization of the domains were found by Pfam and the logo of L1–L4 sequences were obtained in Meme (Bailey et al., 2009; Finn et al., 2014). The dendrograms were created in Mega6 and edited in iTOL (Letunic and Bork, 2007; Tamura et al., 2013). The sequences were aligned by ClustalW2 program and subjected to the tree building process with Maximum Likelihood method by Mega6. The comparison of four laccase-encoding genes was done with K2+G model (Mega6 model test), while the strain tree was done on nucleotide sequence of laccase orthologs (genes encoding for Gr1 and Gr5 proteins) with T92+G model (1000 bootstrap). The orthologs were found by Proteinortho v.5.11 (Lechner et al., 2011) and the chosen genes had the algebraic connectivity of 0.915. Sequence similarities were assessed by SIAS (2015).

### Modeling

Homology models were obtained by I-Tasser (Yang et al., 2015), the analysis of the models were done in pyMOL, ModFOLD4, and WinCoot (McGuffin et al., 2013).

## Results and Discussion

### Redundancy Analysis

Over 300 protein sequences that possess Cu-oxidase domains were downloaded from Broad Institute website. In the first step of the analysis, 214 sequences with MCO1 and MCO2 were retained. The sequences were divided into 20 groups, additionally there were 9 proteins that could not be included in neither of the groups as their identity was much below 95%. In the next step of the analysis, only those proteins were taken into account in which L1 and L3 could be found. Those 80 proteins were divided into 5 groups. Next, the group which does not have the L2 and L4 signature was excluded (Gr3). Deeper analysis was done with proteins from the Step 2.

### Molecular Characteristics

**FIGURE 1:**
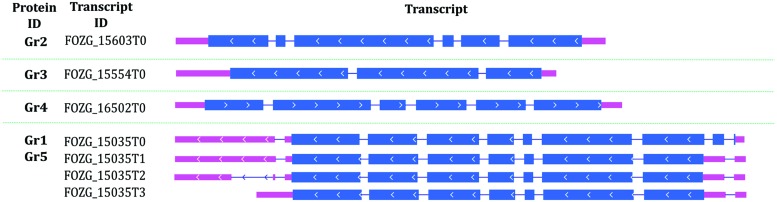
**Putative laccase genes structures on the example of *Fusarium oxysporum* Fo47 genes**.

The length of *F. oxysporum* potential laccase genes varies between 1957 and 2471 nucleotides, while GC content of the genes ranges between 47 and 51% (**Table 2**). There are 2–9 introns in individual genes, which are not distributed similarly, thus division into subgroups within the species cannot be done as proposed in earlier scientific work (Kilaru et al., 2006; Cázares-García et al., 2013). We may observe that Gr3 gene is far distinct from other, which explains the exclusion of Gr3 in the last step of the analysis. Gene encoding for potential laccase Gr1 is the same gene that encodes for Gr5, although with an additional sequence on 5′ end (**Figure 1**).

**Table 2:** Characteristics of putative laccases genes.

Protein	Gene length	cDNA length	No. of introns	GC content (%)
Gr1	2471	2064	9	47
Gr2	2241	1995	5	49
Gr3	1957	1854	2	51
Gr4	2238	1977	5	49
Gr5	2287	1980	6	47

**FIGURE 2:**
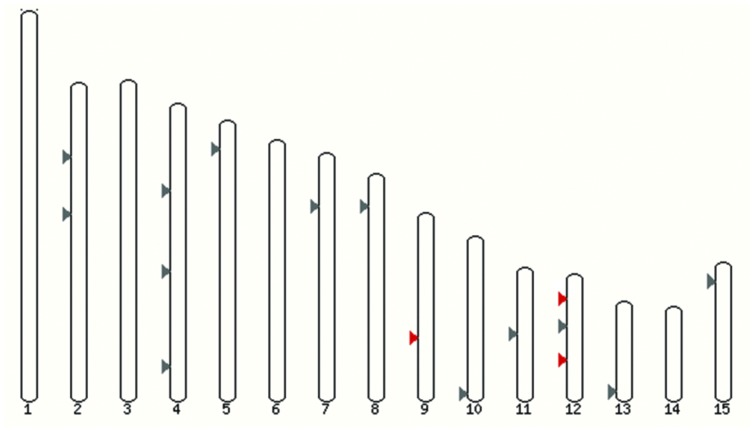
**Localization of laccase genes in *F. oxysporum* f. sp. *lycopersici* 4287 genome.** Grey, all multicopper oxidases genes; red, FOXG_12706 (Chr9), FOXG_13227 (Chr12), and FOXG_14565 (Chr12) – laccases *sensu stricto* (Ensembl).

Annotations of *F. oxysporum* strains genomes are in progress, so far one of 12 genomes is assembled in chromosomes. All potential multicopper oxidase-encoding genes are far from each other and are located on different chromosomes (**Figure 2**). Similar situation was observed before, for example in the genome of *Trichoderma* species or *Laccaria bicolor*; however, clusters of laccase genes were also discovered before (Courty et al., 2009).

The length of chosen proteins varies between 617 and 687 amino acids (**Table 3**), which is atypical for fungal laccases (normally 500–600 amino acids). Putative laccases from *F. oxysporum* seem to have 68–76 kDa, while the typical weight of these protein ranges between 60 and 70 kDa; however, laccases of 70–80 kDa were also reported (Slomczynski et al., 1995; Eggert et al., 1996; Chefetz et al., 1998; Madhavi and Lele, 2009).

**Table 3:** Characteristics of proteins in the groups.

Gr	Length (aa)	pI	MW (kDa)	SL
1	687	5.81	70.8	E
2	664	5.32	75.6	E
3	617	5.66	68.2	T
4	658	6.19	74.8	E
5	659	5.73	75.2	I

*SL, consensus subcellular localization: E, extracellular; T, transmembrane; I, intracellular (ExpasyComputepI/mW; SignalP 4.0, TMHMM2.0, Phobius).*

The consensus subcellular localization was determined for all five groups of laccases using SignalP 4.0, TMHMM2.0, and Phobius. Gr1, 2 are considered to be extracellular laccases (0.480 and 0.514, respectively, according to SignalP); moreover, Gr4 could also be an extracellular protein (0.439). Gr5 must be an intracellular laccase, this protein possess identical amino acid sequences to Gr1, however, without the first 28 amino acids, which is a further sign of the extracellular nature of Gr1 laccase. The putative signal sequence of extracellular laccases were about 25–26 amino acid long. N-glycosylation were predicted by NetNGlyc 1.0. All of them seems to have 11–13 Asn–Xaa–Ser/Thr sequons.

Three Cu-oxidase domains are present in proteins from Gr1–5. The first domain from N-terminus is Cu-oxidase3, then Cu-oxidase and the closest to C-termini Cu-oxidase2, which is characteristic for ascolaccases. Although the proteins from Step 1 possess all three Cu-oxidase domains, their localization on the sequence varies.

**FIGURE 3:**
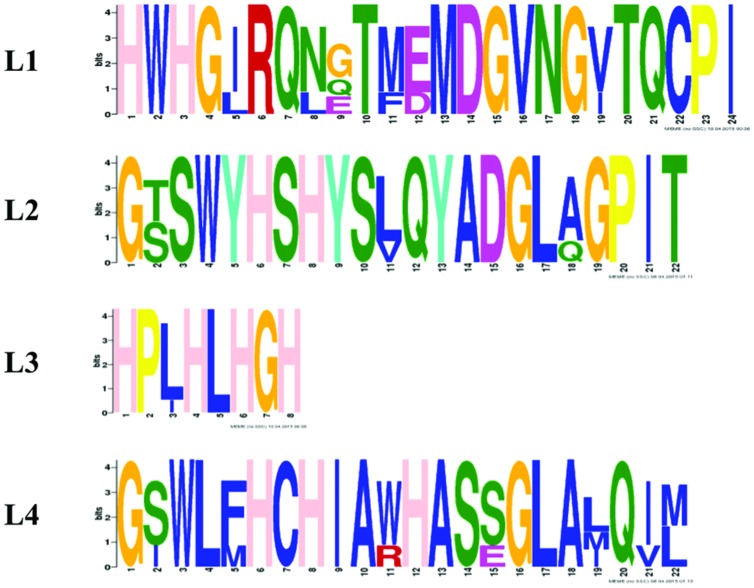
**Logo sequences for Gr1, 2, 4, and 5 for L1–L4 signatures done in Meme**.

**FIGURE 4:**
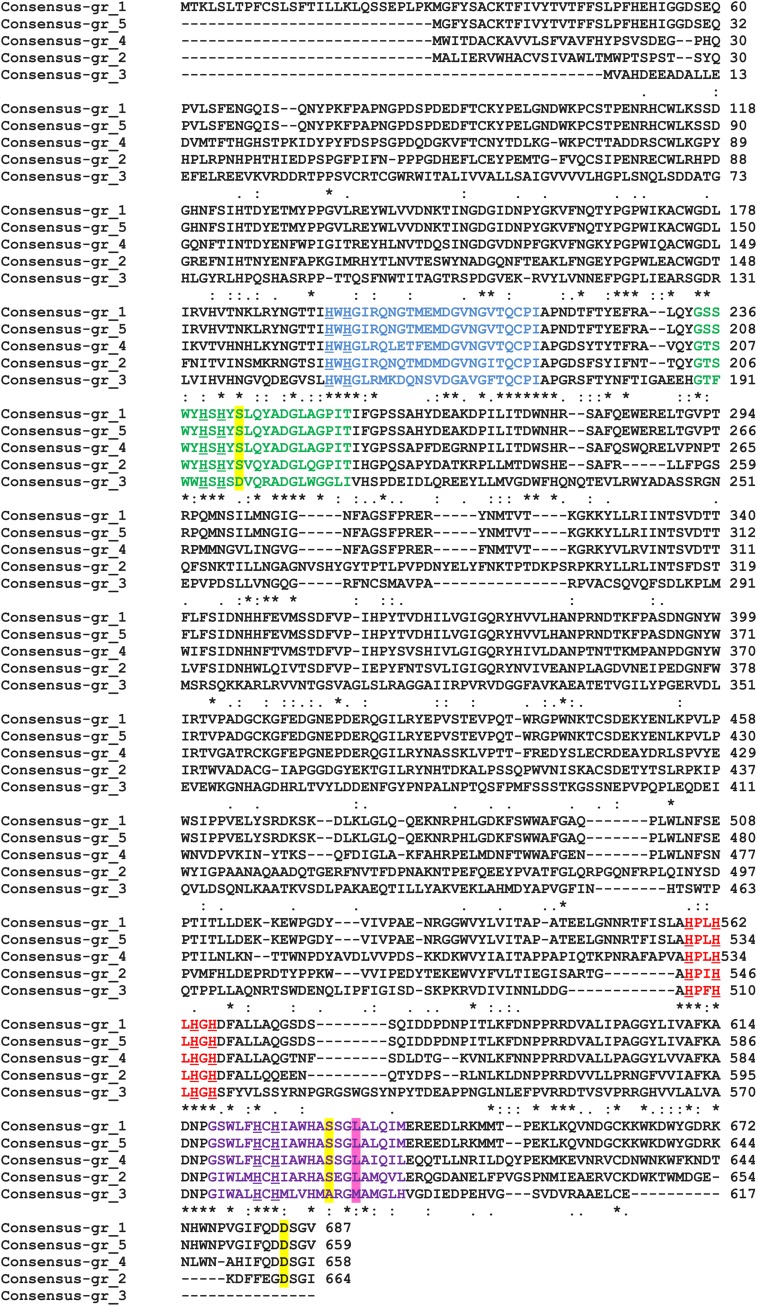
**MSA of five chosen enzymes performed by ClustalW2.** L1, blue; L2, green; L3, red; and L4, violet. H residues involved in copper binding – underlined, SDS-gate – yellow shaded, axial coordination – pink shaded. An asterisk indicates that the residus in a given position are identical, the colon indicates conserved substitutions while a dot signifies semiconserved substitutions.

All proteins from Step 2 and 3 possess L1–L4 laccase signatures; however, Gr3 is relatively distinct from the others. The signatures of laccases from Step 3 are far more conservative than those proposed earlier (Kumar et al., 2003; Cázares-García et al., 2013), in L1 17/24aa are conserved in L2 20/22, in L3 7/9, and in L4 15/22 (**Figure 3**). The signatures are composed of the most important amino acids of laccases, those that coordinates copper atoms. Multiple sequence alignment of chosen protein further reveals differences between Gr3 and other sequences (**Figure 4**). Gr3 somehow lacks the C-termini characteristic for GR1, 2, 4, 5, which signifies that either the D residue forming SDS gate is located in different position or the proton transfer in this protein would function distinctly.

Axial coordination is an important feature that affects redox potential of laccases (Hakulinen et al., 2002; Kumar et al., 2003; Garavaglia et al., 2004; Hakulinen and Rouvinen, 2015; Jones and Solomon, 2015; Mate and Alcalde, 2015). It was suggested that if laccase has Leu or Phe in around tenth position downstream the conserved Cys residue in L4, then the enzyme has a higher *E*_0_ –redox potential (700–800V), whereas the lower *E*_0_ is observed when Met is in this position. Laccases are classified into Lac1 (Met), Lac2 (Leu), and Lac3(Phe) on the basis of axial coordination. This would mean that the chosen putative laccases should be classified as Lac2 (Gr1, 2, 4, 5) proteins and should exhibit higher *E*_0_, while Gr3 should display a lower *E*_0_ (Lac1).

A classification with LccED database was performed with usage of a blast tool available on the website (default settings). With high probability Gr1, 2, 4, and 5 belong to H Family B1 (*Ascomycetes*-like MCO), while Gr4 is classified as H Family A2 (*Basidiomycete* laccases) (Sirim et al., 2011).

**FIGURE 5:**
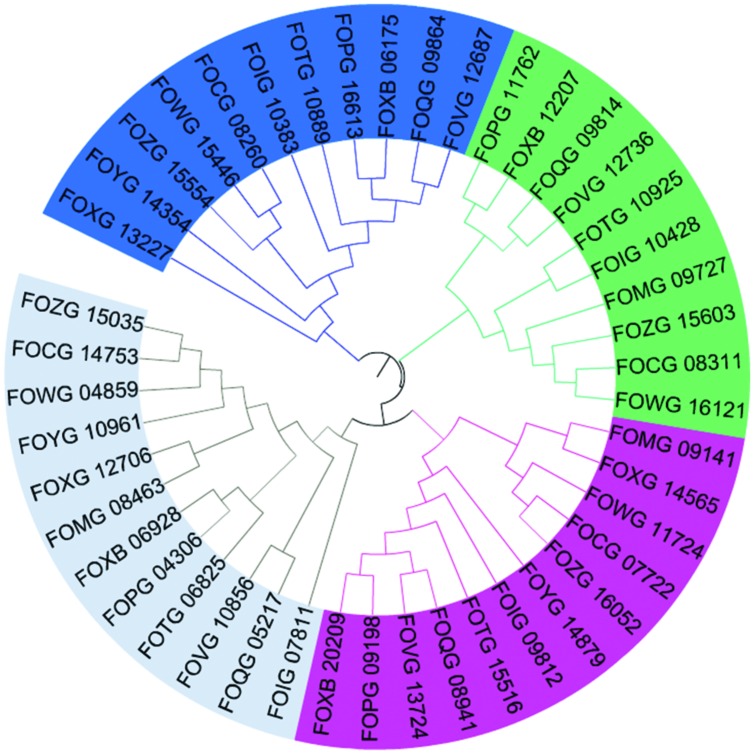
**Phylogenetic tree of all 45 genes that may encode for laccases.** The colors signify division to four groups: grey, Gr1 and Gr5; green, Gr2; blue, Gr3; and pink, Gr4 (Mega6, iTOL).

Multiple sequence alignments and dendrogram formation were essential for the redundancy analysis. The **Figure 5** presents a dendrogram of all genes from Step 2 analysis, which visualize clearly the relationship between all four chosen genes. The identities between the 20 groups from Step 1 ranges between 20 and 97%. The groups with the highest identity are exactly Gr1 and Gr5 from Step 2 which differ only with the signal peptide in Gr1 protein.

**FIGURE 6:**
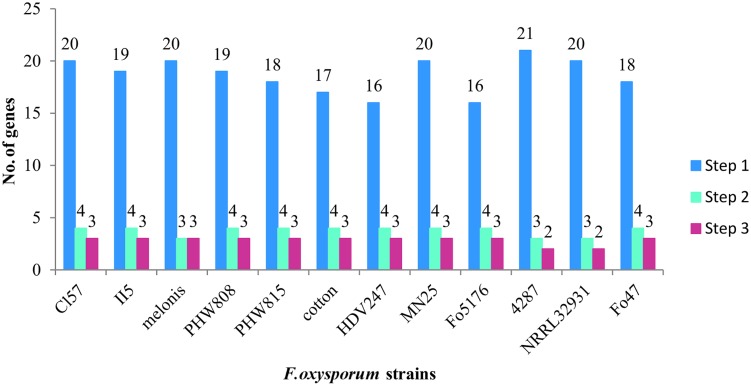
**Number of genes coding for multicopper oxidases (Step 1), multicopper oxidases with certain features of laccases (Step 2), and laccases *sensu stricto* (Step 3)**.

**FIGURE 7:**
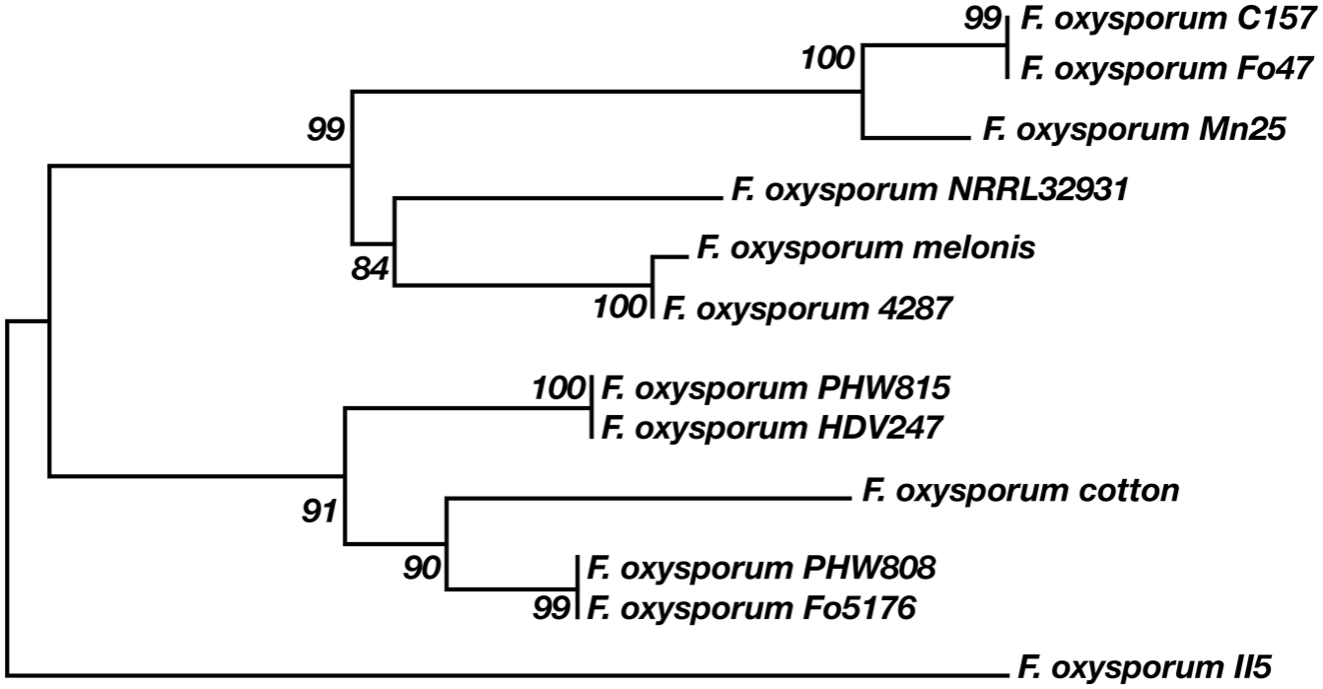
**Relationship between 12 *F. oxysporum* strains and laccases**.

According to our analysis, the 12 *F. oxysporum* strains possess in minimum 16–21 potential multicopper oxidase genes and at least two putative laccase genes (**Figure 6**). Twelve *F. oxysporum* strains are similar in context of laccase genes; however, three of them seem to diverge evolutionary from the rest. *F. oxysporum melonis* does not contain a gene encoding for the putative transmembrane Gr3, while *F. oxysporum* f. sp. *lycopersici*4287 lost three putative laccase genes. The human pathogen *F. oxysporum* NRRL32931 lacks Gr1- and Gr2-encoding genes, those that may encode for extracellular proteins, probably due to the fact that it does not have to deal with lignocellulose during its virulence. These three *F. oxysporum* strains form a subgroup on a strain tree presented in the **Figure 7**. The mentioned subgroup is a part of one of three clades that forms the strain tree. Interestingly, the gene from *F. oxysporum* II5, pathogenic toward banana, has the highest number of mutations and was not included in a clade with other strains. *F. oxysporum* II5 as the only strain that was isolated in Indonesia, which climate differ significantly from weather conditions in Europe (Fo47, PHW815), America (Cl57, Mn25, melonis, PHW808), or Australia (HDV247). The climate may be an important factor for strain evolution.

### Modeling

Three homologous models of potential laccases were successfully created by I-Tasser server for Gr2, 3, and 4; Gr1 and 5 models were built with an unsatisfactory effect. For protein Gr2 and 4 a potential signal peptides were removed and the modeling was done again which led to better results. This is a further sign of the extracellular nature of Gr2 and 4 proteins. The models are assessed by ModFOLD4 server as good models with less that 1:1000 chance that the models are incorrect. All three models have problematic region on their N-termini. It may suggest that this region may be cleft off during maturation of the protein (pro-peptide) and that is why the I-Tasser server could not find a match in the PDB database. Such pro-sequences important in protein expression were detected for example in *Melanocarpus albomyces* laccase (Bulter et al., 2003; Kiiskinen and Saloheimo, 2004; Kiiskinen et al., 2004). However, ProP1 server did not discover pro-peptide cleavage sites in these proteins. The Ramachandran plots for the models show a relatively high number of amino acids in disallowed regions (∼17%), which is an indication for further model optimizations.

It was suggested that the 3D position of C-terminus of laccase is important for its activity. C-terminus may act as a plug that obturates the trinuclear (T2/T3) channel, thus preventing oxygen to enter the channel and water to exit it. The trinuclear channel is widely regarded as the oxygen channel, however, a mutant of *M. albomyces* laccase (PDB code 3QPK) suggest a different oxygen route. The models of Gr2 and 4 reveals similar location of C-terminus as observed in *M. albomyces* laccase (2Q90; Kiiskinen and Saloheimo, 2004; Bleve et al., 2013, 2014). Such a conformation was proposed to be a common feature of ascolaccases. However, the amino acid sequences of Gr2 and 4 lacks the additional 14 amino acids upstream DSG[LIV] motif which is present in *M. albomyces* laccase. It is hypothesized that incorrect processing of this sequence leads to lack of enzyme activity. Among ascomycetes many laccases share similar C-terminal sequence with *M. albomyces*, which is cleft on a conserved site, while other are similar to our proteins and their sequence is finished with DSG[LIV] motif (*Botrytis cinerea*, *Trichoderma reesei*). It was suggested many times that DSG[LIV] is a common conservative motif for all ascomycetes laccase, which is a further reason for excluding Gr3 protein from laccases *sensu stricto* group (Andberg et al., 2009; Cázares-García et al., 2013). The studies on *M. albomyces* laccase proved that the deletion of DSG[LIV] inactivates the enzyme, whereas the point mutation in this region leads to lower thermostability, turnover number and the structural changes in T2 centre.

**FIGURE 8:**
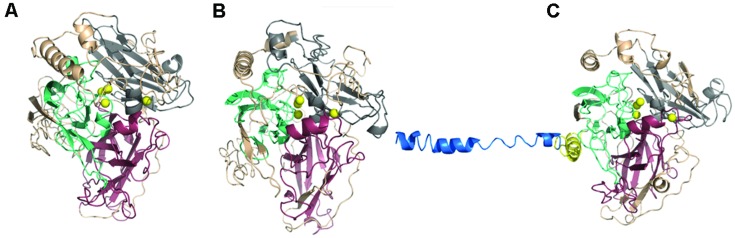
**Gr2 (A), Gr4 (B), and Gr3 (C) models created by I-Tasser.** Pink, Cu-oxidase domain; grey, Cu-oxidase2 domain; cyan, Cu-oxidase3 domain; and Cu atoms (yellow) in an approximate location. Yellow cartoon in **(C)** represents transmembrane region of the protein, blue cartoon represents the outside part.

**FIGURE 9:**
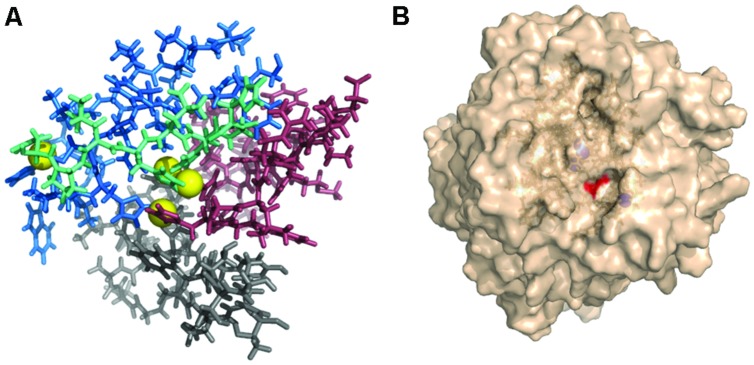
**(A)** L1–L4 signatures encirculating Cu atoms (yellow) (Gr4 model). Pink, L1; grey, L2; cyan, L3; and blue, L4. **(B)** Surface of Gr4 model with DSGI C-terminus sequence shown in red. Cu atoms shown in blue (approximate location).

All modeled proteins hold characteristic 3D architecture. The domains are arranged in a typical greek-key motifs, the L1–L4 signatures encircle Cu atoms. A mononuclear site is located in Cu-oxidase2 domain (third domain from N-terminus), while trinuclear site is between Cu-oxidase3 and Cu-oxidase2 domains (first and third domains from N-terminus) (Hakulinen and Rouvinen, 2015; Pardo and Camarero, 2015). The architecture of Cu centers is similar to those in other studied laccases. T1 copper ion is coordinated by two His and one Cys residue, while T2/T3 center by eight His residues (**Figures 8** and **9**). The substrate binding site is located close to the mononuclear site, close to Cu-oxidase3 domain, whereas oxygen binding pocket is situated at the trinuclear copper site.

## Summary

Four proteins extracted in *in silico* analysis possess all characteristics of laccases *sensu stricto*. Their sequences contain L1–L4 signatures, three Pfam domains and conserved C-terminus. Their structure, according to homologous models, is similar to structures of known laccases. The rest of over 300 genes were not assigned as laccase-encoding genes due to the lack of mentioned sequences. However, further analysis of the proteins is important in their assessment to specific enzyme groups.

Genome-wide structural and phylogenetic *in silico* analysis of laccase genes in a plant pathogen is essential for further research on the virulence of the fungi. The determination of laccases *sensu stricto* may help, among others, in planning of knock-out studies in order to confirm or deny the role of laccase in pathogenicity. Six potential laccase gene were studied by a Spanish team for its role in virulence in tomato (Cañero and Roncero, 2008). Three of them were detected by RT-PCR as expressed during plant infection. *Lcc1*(EF990894.1), *lcc3*(EF990899.1), and *lcc5*(EF990897.1) lacking mutants were created and checked for virulence in root infection assays, leading to the same results as for the wild type strain. However, this negative results does not mean that laccases do not take part in the pathogenicity of *F. oxysporum*. The mentioned putative laccases were not included in our Step 2 and Step 3 analysis because of the lack of as conservative L1–L4 signatures as proposed earlier, thus they may not be laccases *sensu stricto*. Further research is essential for full understanding of laccase role in pathogenicity.

Laccase is nowadays a popular enzyme in many biotechnological applications, scientists and industrial partners still work hard to find laccases with better catalytic efficiencies, hence discovery of novel enzymes is needed. The presented putative laccases, to our knowledge, do not have very close homologs already used in industrial applications. Blastp (default settings) analysis of consensus sequences did not reveal any studied laccases that are identical at least in 50%. This and the fact that these enzymes may have high redox potential due to leucine in axial coordination make the effort of their study more meaningful in the perspective of biotechnological applications.

## Conflict of Interest Statement

The authors declare that the research was conducted in the absence of any commercial or financial relationships that could be construed as a potential conflict of interest.
