# Supplementary material for: Diversity of laccase-coding genes in Fusarium oxysporum genomes
Source: Front Microbiol. 2015 Sep 10;6:933. doi: 10.3389/fmicb.2015.00933 (PMC4564759; doi:10.3389/fmicb.2015.00933)
Supplement: Supplementary file 1 [file Data_Sheet_1.DOCX]

***Supplementary Material***

**Diversity of laccase coding genes in *Fusarium oxysporum* genomes**

Natalia Kwiatos*, Małgorzata Ryngajłło, Stanisław Bielecki

Institute of Technical Biochemistry, Faculty of Biotechnology and Food Sciences, Lodz University of Technology, Lodz, Poland

*Correspondance: Natalia Kwiatos, Institute of Technical Biochemistry, Faculty of Biotechnology and Food Sciences, Lodz University of Technology, Stefanowskiego 4/10, 90-924, Lodz, Poland, [natalia.kwiatos@dokt.p.lodz.pl](mailto:natalia.kwiatos@dokt.p.lodz.pl)

1. **Supplementary figures**

**
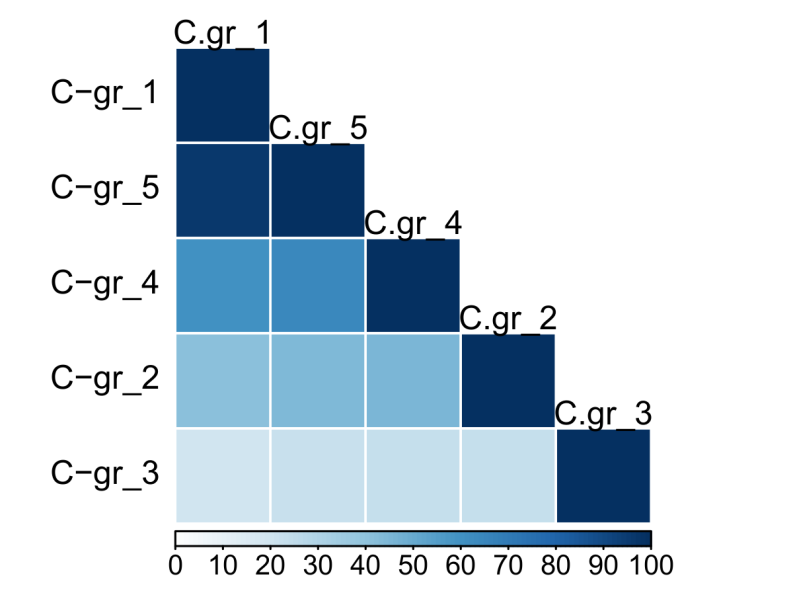
**

**Supplementary Figure 1.** Graphical representation of protein sequence identities of five putative laccases from *F.oxysporum*





**Supplementary Figure 2.** A tree presenting relationship between 5 putative laccases from *F.oxysporum* (ML, Mega6)

**Supplementary Table 1.** Numbers of multicopper oxidase genes in *F.oxysporum* strains

| **Strain ID** | **Strain Name** |  | **Potential genes encoding for MCO domains (Step 1)** | **Potential genes encoding for laccases *sensu stricto* (Step 2)** | **Potential genes encoding for laccases *sensu stricto* (Step 3)** |
| --- | --- | --- | --- | --- | --- |
| FOCG | Cl57 |  | 20 | 4 | 3 |
| FOIG | II5 |  | 19 | 4 | 3 |
| FOMG | 26406 |  | 20 | 3 | 3 |
| FOPG | PHW808 |  | 19 | 4 | 3 |
| FOQG | PHW815 |  | 18 | 4 | 3 |
| FOTG | cotton |  | 17 | 4 | 3 |
| FOVG | HDV247 |  | 16 | 4 | 3 |
| FOWG | MN25 |  | 20 | 4 | 3 |
| FOXB | Fo5176 |  | 16 | 4 | 3 |
| FOXG | 4287 |  | 21 | 3 | 2 |
| FOYG | NRRL32931 |  | 20 | 3 | 2 |
| FOZG | Fo47 |  | 18 | 4 | 3 |
